# The correlation between hepatic controlled attenuation parameter (CAP) value and insulin resistance (IR) was stronger than that between body mass index, visceral fat area and IR

**DOI:** 10.1186/s13098-024-01399-5

**Published:** 2024-07-09

**Authors:** Zhouhuiling Li, Renjiao Liu, Xinying Gao, Dangmin Hou, Mingxin Leng, Yanju Zhang, Meiyang Du, Shi Zhang, Chunjun Li

**Affiliations:** 1 Tianjin University of Traditional Chinese Medicine, Tianjin, China; 2 Department of Endocrinology, Health Management Center, Tianjin Union Medical Center, Nankai University Affiliated Hospital, Tianjin, China; 3 Tianjin Medical University, Tianjin, China

**Keywords:** Obesity, Insulin resistance, Controlled attenuation parameter

## Abstract

**Background:**

Hepatic controlled attenuation parameter (CAP) is a novel marker for quantifying hepatic fat accumulation. Insulin resistance (IR) plays a major role in the pathogenesis and natural history of hepatic steatosis. This study aimed to investigate the possible relationship between CAP value and IR.

**Methods:**

This study included a total of 420 patients with overweight or obesity who came to the obesity clinic at Tianjin Union Medical Center. Vibration-controlled transient elastography examination was conducted to detect CAP and liver stiffness measurement (LSM) values. Body composition, including visceral fat area (VFA), and body fat mass (BFM), was evaluated by the direct segmental multi-frequency bioelectrical impedance analysis (BIA). The associations between CAP value, body mass index (BMI), VFA, BFM and homeostasis model assessment of insulin resistance (HOMA-IR) were analyzed.

**Results:**

CAP value was positively associated with HOMA-IR (r = 0.568, P < 0.001), the strength of which was much stronger than BMI, VFA, and BFM. In multivariate linear regression, CAP value and HOMA-IR showed a significant positive association (adjusted β = 0.015, 95% CI 0.007–0.022,* P* < 0.001). Subgroup analysis suggested no significant interaction between CAP value and HOMA-IR across age, BMI, LSM, hypertension, and sex groups (all *P* for interaction > 0.05).

**Conclusions:**

Hepatic CAP value is more remarkably than other obesity markers associated with HOMA-IR in individuals with overweight or obesity, regardless of age, BMI, LSM, hypertension, and sex.

**Supplementary Information:**

The online version contains supplementary material available at 10.1186/s13098-024-01399-5.

## Introduction

Obesity can lead to a series of metabolic diseases, including type 2 diabetes mellitus (T2DM), metabolic dysfunction–associated steatotic liver disease (MASLD) and metabolic syndrome [1, 2]. According to the most recent national survey, more than half of Chinese adults are with obesity or overweight [3], which is a major public health issue in China. In individuals with obesity, ectopic fat deposition typically begins in the liver, which is essential for glucose-lipid metabolism [4, 5]. Excess energy intake can cause hepatic fat deposition, and eventually lead to hepatic steatosis. If the amount of hepatic steatosis exceeds 5%, it is known as MASLD, which reflects an early stage of obesity [6].

Insulin resistance (IR) plays a major role in the pathogenesis and natural history of MASLD. IR is a pathological condition where the body's sensitivity to insulin is reduced, leading to diminished glucose utilization and abnormal lipid metabolism [7, 8]. Once an individual is diagnosed with obesity, the enlargement of adipose tissue and the appearance of ectopic fat deposits cause the body to be inflammatory. A long-term low-grade inflammatory state prevents insulin from its action in the insulin signaling pathway, leading to IR. Then IR further contributes to hepatic de novo lipogenesis and deposition of ectopic fat [9]. As such, IR is one of the most important causes of MASLD, identifying and treating IR in people with obesity is critical in avoiding the incidence and development of metabolic diseases. Homeostasis model assessment of insulin resistance (HOMA-IR) is a recognized model for assessing IR, which is simple to operate and virtually non-invasive to patients. We hypothesized that there might be an association between hepatic steatosis and HOMA-IR in populations with overweight or obesity.

Hepatic controlled attenuation parameter (CAP) value is a novel marker for quantifying hepatic fat accumulation. A study [10] found that adiposity and the severity of IR are the main determinants of CAP value even among individuals with metabolic dysfunction. However, few studies have discussed the direct relationship between CAP value and HOMA-IR. Our study aimed to determine whether there is an association between CAP value and HOMA-IR in populations with overweight or obesity.

## Methods

### Study design and participants

The study participants were patients who came to the obesity clinic at Tianjin Union Medical Center from January 2021 to February 2024. The inclusion criteria included: (1) patients over the age of 18 years, (2) patients with a body mass index (BMI) of 24 kg/m^2^ or above, (3) patients who do not receive insulin therapy or any oral medication that affects fasting insulin (FINS). Among the 929 patients who met the inclusion criteria, we further excluded: (1) pregnant women, people taking oral contraceptives and hormones, and those who were detected as having oncological, infectious, hyperthyroidism, or serious liver or renal disease, (2) weekly alcohol intake 140–350 g female, 210–420 g male (average daily 20–50 g female, 30–60 g male), (3) missing indicator value. Ultimately, 420 study participants were included in our study. The study was approved by the Medical Ethics Committee of Tianjin Union Medical Center (No. 2021C06), and all participants provided informed consent before participating in the study.

### Variables and data measurement

All data in this study were collected and recorded by uniformly trained researchers. Demography and clinical information were recorded for each participant.

#### Measurement of hepatic steatosis

Hepatic fat accumulation was quantified using vibration controlled transient elastography. Measurements were taken on subjects in the dorsal recumbent position using a FibroScan 502 Touch (Echosens, Shenzhen, China) with the XL probe at the optimal measurement point previously defined by a morphological US (good acoustic window and no blood vessel in the area). The data was stored and transferred to a computer for processing and extraction of CAP and liver stiffness measurement (LSM) values from the FibroScan output. The grade of hepatic fat accumulation was defined according to the CAP value [11]: S0 (steatosis ≤ 10%, CAP value ≤ 238), S1 (steatosis ≥ 11%, 238 < CAP value ≤ 259), S2 (steatosis ≥ 34%, 259 < CAP value ≤ 292), and S3 (steatosis ≥ 67%, CAP value > 292). The manufacturer of FibroScan provided the cut-offs for grading.

#### Measurement of IR

HOMA-IR received recognition as a model to reflect IR better and is suitable for epidemiological research studies. The formula is as follows: fasting blood glucose (FPG, mmol/L) × fasting insulin (FINS, μU/mL)/22.5 [12]. Fasting blood glucose was measured by the glucose oxidase method, and fasting insulin was measured by chemiluminescent immunoassay.

#### Anthropometric and body composition

All participants had their anthropometric measurements, including weight and height, measured under fasting conditions, wearing light clothes and no shoes. Participants stood in the position marked by the scale and kept their heads, hips, and feet in a straight line. Height and weight were measured using the Ultrasonic Height and Weight Scale HNH-219 (OMRON, Shenzhen, China). BMI was calculated as weight (kg)/height (m) squared. Body composition including visceral fat area (VFA), body fat mass (BFM), soft lean mass (SLM), and percent of body fat (PBF) measured with the direct segmental multi-frequency bioelectrical impedance analysis method (Inbody 770, Biospace Co., BR-Chinese-C7-B-140218).

#### Biochemical assessments

Biochemical indicators included FINS, FPG, hemoglobin A_1c_ (HbA_1c_), uric acid (UA), total cholesterol (TC), triglyceride (TG), high‐density lipoprotein cholesterol (HDL-C), low-density lipoprotein cholesterol (LDL-C), gamma-glutamyl transpeptidase (GGT), alanine aminotransferase (ALT), and aspartate aminotransferase (AST). The blood samples were collected in the morning after an overnight fast, immediately centrifuged, and stored at -80℃ for subsequent detection assays. An automatic biochemical analyzer (TBA-120FR, Toshiba, Japan) was used to determine the levels of all biochemical indicators.

### Statistical analysis

All statistical analyses were performed using SPSS Statistics 26 and GraphPad Prism 8. *P* value < 0.05 (bilateral) indicates statistical significance. The baseline table of the study population was statistically described by steatosis groups based on CAP value. For descriptive statistics, continuous variables were reported as means (standard deviation [SD]), and median (interquartile ranges [IQRs]); categorical variables were presented as counts (percentages). Between-group comparisons of demographic and clinical characteristics were performed using Student’s t-test, Mann–Whitney Wilcoxon test, one-way ANOVA, or Chi-square tests as appropriate. Spearman's correlation analysis was adopted to investigate the correlation between obesity markers and HOMA-IR. The β values and 95% confidence intervals were calculated using univariate linear regression analysis and multivariate linear regression analysis.

In order to investigate confounders, we added or removed covariates one by one in the linear regression model for correction and compared the corresponding effect values. Besides age and sex, covariates with more than a 10% change in effect values were selected for multi-model correction. During covariate selection, a variance inflation factor ≥ 5 indicated the presence of multicollinearity, weight and BFM were excluded. FPG and FINS were highly associated with IR and excluded. Age, sex, BMI, VFA, HbA_1c_, and ALT were ultimately included in the multi-model correction. Finally, we performed interaction and univariate linear regression analyses according to age (< 40 or ≥ 40 years), BMI (< 30 or ≥ 30 kg/m^2^), LSM (< 7.9 or ≥ 7.9 kPa), hypertension (no or yes), and sex (male or female).

## Results

### Description of basic information about participants

**Fig. 1:**
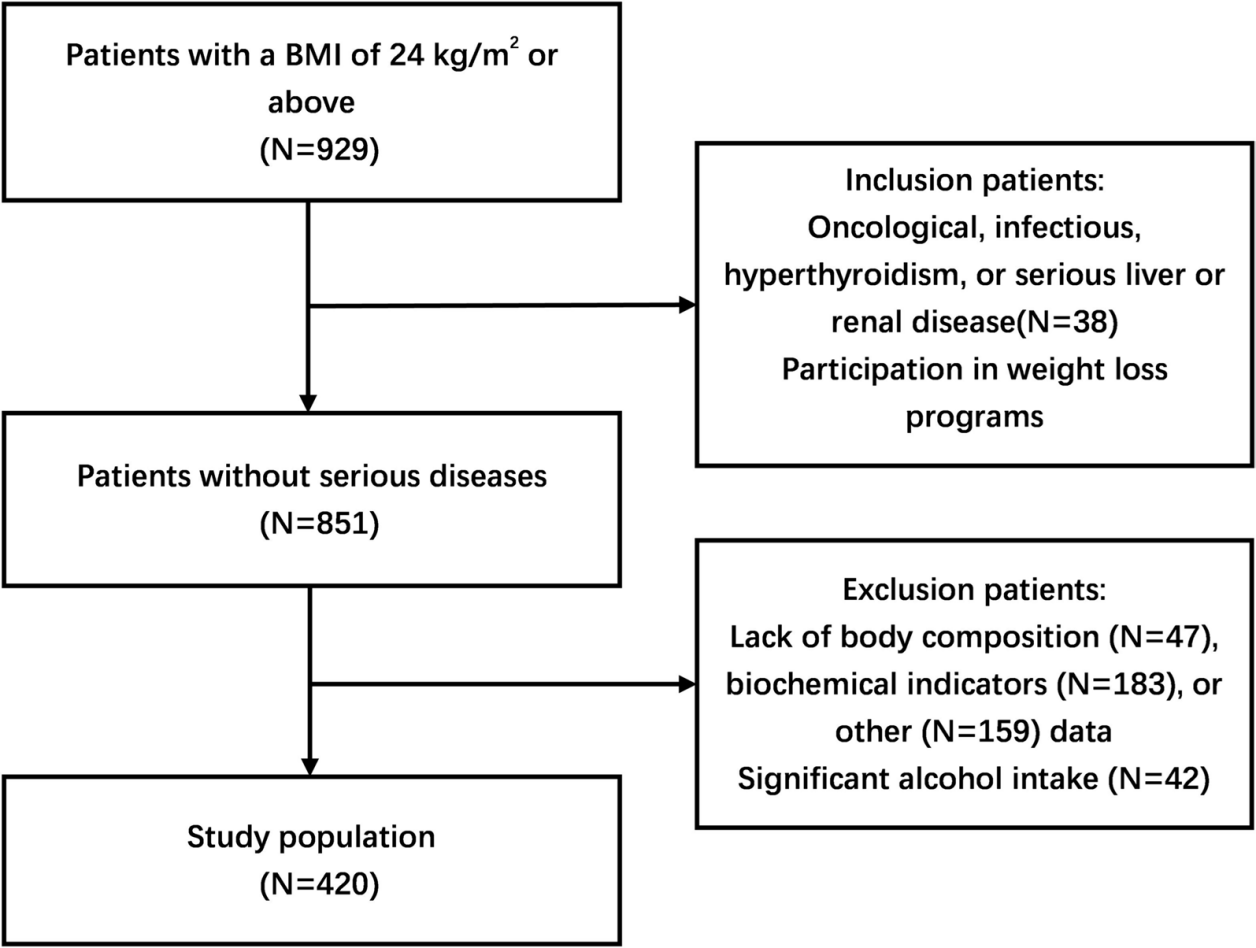
Flowchart for the patient recruitment. BMI, body mass index

Between January 2021 and February 2024, we assessed 929 potentially eligible participants, 420 (123 men and 297 women) of whom were included in the study. The flowchart of participant selection is depicted in Fig. 1.

The groups were graded according to CAP value and the characteristics of the participants are presented in Table S1 [see Additional file 1]. The participants had a mean ± standard deviation (SD) age of 34.42 ± 9.90 years and a height of 166.84 ± 8.52 cm. Significant statistical differences were detected in sex, height, weight, BMI, SBP, DBP, CAP, LSM, VFA, BFM, SLM, PBF, FPG, FINS, HOMA-IR, HbA_1c_, UA, TC, TG, HDL-C, LDL-C, GGT, ALT, AST and prevalence of hypertension among different groups (all *P*-values < 0.05). In comparison to other groups, S3 (severe steatosis group) had a higher proportion of hypertension, with higher levels of weight, SBP, DBP, LSM, BMI, VFA, BFM, SLM, PBF, FPG, FINS, HbA_1c_, UA, TC, TG, LDL-C, GGT, ALT, AST and HOMA-IR but lower levels of direct HDL-C (all *P* for trend < 0.05). Notably, participants with higher CAP values tended to have higher HOMA-IR.

### Association between obesity markers and HOMA-IR

**Fig. 2:**
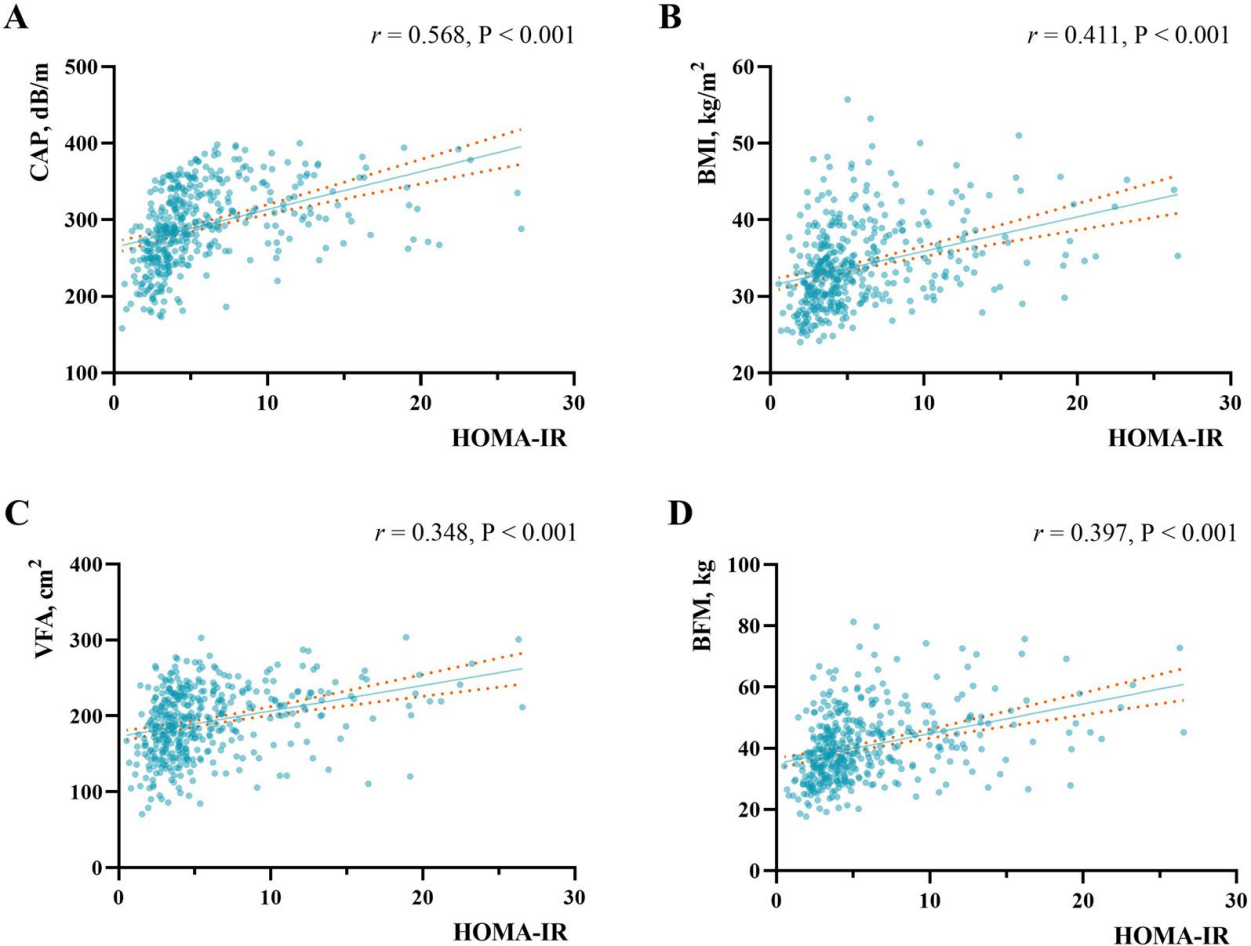
Correlation between HOMA-IR and obesity markers. **A** Correlation between HOMA-IR and CAP value. **B** Correlation between HOMA-IR and BMI. **C** Correlation between HOMA-IR and VFA. **D** Correlation between HOMA-IR and BFM. Each point represents a sample. The solid line represents the smooth curve fit between variables. The dotted line represents the 95% confidence interval from the fit. HOMA-IR, homeostasis model assessment of insulin resistance; CAP, controlled attenuation parameter; BMI, body mass index; VFA, visceral fat area; BFM, body fat mass

We analyzed correlations between obesity markers and HOMA-IR in the population with overweight or obesity. Figure 2 shows that CAP, BMI, VFA, and PBF were all positively and linearly correlated with HOMA-IR. There was a moderate correlation between CAP value and HOMA-IR of available histologic samples with a Pearson correlation coefficient of 0.568 (*P* < 0.001). The correlation between BMI and HOMA-IR of available histological samples had a Pearson correlation coefficient of 0.411 (*P* < 0.001). The correlation coefficient between VFA and HOMA-IR of available histological samples was 0.348 (*P* < 0.001), and the correlation coefficient between PBF and HOMA-IR for the available histological samples was 0.397 (*P* < 0.001). Notably, the CAP value showed a positive association with HOMA-IR, much stronger than that of BMI, VFA, and BFM.

### Univariate and multivariate analyses of CAP and HOMA-IR

As shown in Table 1, the univariate analysis indicated that CAP, age, height, weight, BMI, VFA, BFM, SLM, PBF, SBP, DBP, HbA_1c_, UA, TC, TG, HDL-C, LDL-C, GGT, ALT, AST, and LSM were associated with HOMA-IR (all *P*-values < 0.05). HOMA-IR levels increased by 0.033 (95% CI 0.026 to 0.041) for every unit increase in CAP value.

**Table 1:** Results of univariate analysis of HOMA-IR

Variables	β (95%CI)	P-value
CAP, (dB/m)	0.033 (0.026, 0.041)	< 0.001
Age, (years)	−0.058 (−0.099, −0.018)	0.005
Height, (cm)	0.088 (0.041, 0.135)	< 0.001
Weight, (kg)	0.087 (0.067, 0.107)	< 0.001
BMI, (kg/m^2^)	0.269 (0.200, 0.339)	< 0.001
VFA, (cm^2^)	0.030 (0.021, 0.039)	< 0.001
BFM, (kg)	0.132 (0.099, 0.165)	< 0.001
SLM, (kg)	0.112 (0.075, 0.150)	< 0.001
PBF, (%)	0.095 (0.035, 0.155)	0.002
SBP, (mmHg)	0.073 (0.049, 0.097)	< 0.001
DBP, (mmHg)	0.095 (0.062, 0.128)	< 0.001
HbA1c, (%)	2.268 (1.866, 2.670)	< 0.001
UA, (μmol/L)	0.006 (0.002, 0.010)	0.007
TC, (mmol/L)	0.520 (0.198, 0.843)	0.002
TG, (mmol/L)	0.928 (0.600, 1.255)	< 0.001
HDL-C, (mmol/L)	−3.569 (−4.910, −2.228)	< 0.001
LDL-C, (mmol/L)	0.834 (0.299, 1.368)	0.002
GGT, (U/L)	0.044 (0.029, 0.059)	< 0.001
ALT, (U/L)	0.039 (0.028, 0.050)	< 0.001
AST, (U/L)	0.070 (0.047, 0.092)	< 0.001
LSM, (kPa)	0.331 (0.230, 0.433)	< 0.001

*HOMA-IR* homeostasis model assessment of insulin resistance, *CAP* controlled attenuation parameter, *BMI* body mass index, *VFA* visceral fat area, *BFM* body fat mass, *SLM* soft lean mass, *PBF* percent body fat, *SBP* systolic blood pressure, *DBP* diastolic blood pressure, *LSM* liver stiffness measurement, *HbA*_1c_ hemoglobin A_1c_, *UA* uric acid, *TC* total cholesterol, *TG* triglyceride, *HDL-C* high‐density lipoprotein cholesterol, *LDL-C* low-density lipoprotein cholesterol, *GGT* gamma-glutamyl transpeptidase, *ALT* alanine aminotransferase, *AST* aspartate aminotransferase

We conducted a multivariate linear regression analysis. Table 2 shows the results of the multivariate regression analysis to reveal the inherent patterns between the elements further. All models controlling for potential confounders showed a positive correlation between CAP value and HOMA-IR. In model 1, we adjusted for age, sex, and BMI, CAP value per one-unit rise was highly associated with HOMA-IR (β = 0.026, 95% CI 0.018 to 0.035, *P* < 0.001). After adjusting for model 1 and VFA, the positive correlation became more significant in model 2 (β = 0.026, 95% CI 0.018 to 0.035, *P* < 0.001). After adjusting for all covariates, the relationship between CAP value and HOMA-IR became weaker in model 3 (β = 0.015, 95% CI 0.007 to 0.022, *P* < 0.001).

**Table 2:** Multivariable-adjust β and 95%CI of the CAP value associated with HOMA-IR

	R^2^	β (95%CI)	t	P-value
Unadjusted	0.165	0.033 (0.026, 0.041)	9.102	< 0.001
Model1	0.204	0.026 (0.018, 0.035)	6.209	< 0.001
Model2	0.209	0.026 (0.018, 0.035)	6.173	< 0.001
Model3	0.376	0.015 (0.007, 0.022)	3.620	< 0.001

Model 1 adjusts for age, sex, and BMI. Model 2 adjusts for Model 1 + VFA. Model 3 adjusts for Model 1 + Model 2 + HbA_1c_ and ALT

*CAP* controlled attenuation parameter, *HOMA-IR* homeostasis model assessment of insulin resistance, *BMI* body mass index, *VFA* visceral fat area, *HbA1c* hemoglobin A_1c_, *ALT* alanine aminotransferase

### Subgroup analysis

**Fig. 3:**
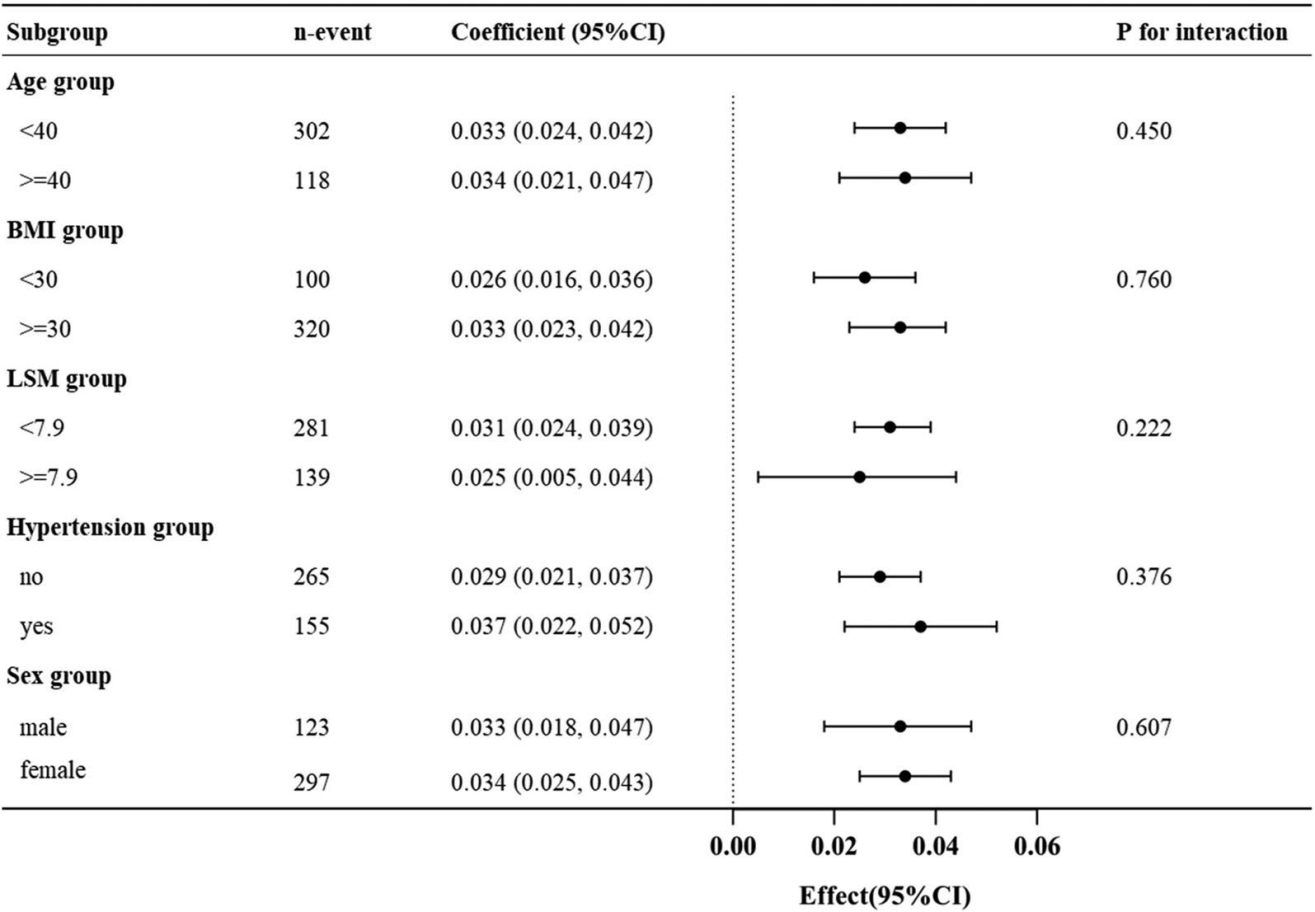
Subgroup analysis of the association between CAP value and HOMA-IR. CAP, controlled attenuation parameter; HOMA-IR, homeostasis model assessment of insulin resistance; BMI, body mass index; LSM, liver stiffness measurement

We did a subgroup analysis by age (< 40 or ≥ 40 years), BMI (< 30 or ≥ 30 kg/m^2^), LSM (< 7.9 or ≥ 7.9 kPa), hypertension (no or yes), and sex (male or female) to survey if the results did not apply to the different population with overweight or obesity. The results of the subgroup analyses are shown in Fig. 3. The relationship between CAP value and HOMA-IR remained stable across all subgroups, including age (*P* for interaction = 0.450), BMI (*P* for interaction = 0.760), LSM (*P* for interaction = 0.222), hypertension (*P* for interaction = 0.376), and sex (*P* for interaction = 0.607).

## Discussion

This study found that a higher level of CAP value was independently associated with a higher risk of IR in a Chinese population with overweight or obesity. Besides, the associations remained similar in the subgroups stratified by liver fibrosis. The correlation between CAP value and HOMA-IR was notably stronger than BMI, VFA, and BFM. Our study revealed a moderate positive correlation between CAP value and HOMA-IR in linear regression analysis, which suggested CAP value may be associated with the severity of IR.

In 2010, Sasso [13] et al. first reported a new method for evaluating hepatic steatosis, the CAP value. This method has gained popularity in the noninvasive diagnosis of steatotic liver disease because it can quantitatively measure hepatic steatosis with a fat content of 10% or more, and has good sensitivity and specificity [6, 14–16]. As the gold standard for quantifying hepatic steatosis, magnetic resonance imaging proton density fat fraction (MRI-PDFF) provides more accurate quantification of hepatic fat content [17], but it lacks universality because of its high cost and limited availability. Furthermore, abdominal ultrasonography is a non-invasive, convenient, and inexpensive method for detecting hepatic steatosis and its degree. Abdominal ultrasonography diagnosis of steatotic liver disease and its degree is, however, influenced by the subjective judgement of the doctor. In comparison, CAP has recently become more desirable for clinical practice and research. As far as we know, this study is the first one to use CAP value to evaluate hepatic steatosis and investigate its correlation with IR.

In terms of the relationship between hepatic steatosis and IR, previous studies have shown different views. Wei. Y et al. have found positive associations between Adipo-IR and MASLD in Chinese adults, especially in postmenopausal women with hyperlipidemia [18]. The research conducted by Luukkonen PK showed that ketogenic diet could reduce intrahepatic triglycerides in obese participants by improving hepatic IR [19]. Numerous cohort studies and clinical randomized trials have shown that reducing hepatic fat accumulation through dietary or exercise interventions is accompanied by an improvement in IR [20–22]. Hepatic steatosis was defined by abdominal ultrasonography or MRI in these studies and their results are consistent with ours. CAP can accurately quantify the content of hepatic steatosis compared with these methods. Conversely, a cross-sectional study, carried out by Bril, F, et al. and performed on 352 healthy individuals, showed that hepatic insulin sensitivity did not decrease further after a threshold of intrahepatic triglycerides accumulation [23]. The opposite result may result from the fact that this study focused on different populations without obesity and hepatic IR usually precedes peripheral IR [24].

Our results showed that the correlation between CAP value and HOMA-IR was stronger than that between other obesity markers (BMI, VFA, and BFM) and HOMA-IR. A study by Hiroshi Yatsuya also showed that hepatic fat was associated with IR independent of age, sex, BMI, PBF, and waist circumference in Japanese adults [25]. Further, some investigators have suggested that most of the metabolic complications of visceral obesity are mediated by the metabolic abnormalities that accompany excess hepatic fat [26], CAP value may indicate metabolic disorders earlier than VFA. A study conducted in Japan suggested that hepatic fat may be a more useful clinical marker than VFA to predict IR in Japanese men without obesity and diabetes [27]. Another cross-sectional study claimed that steatotic liver was a risk factor for T2DM and liver fibrosis in Japanese, but VFA was not [28]. It has been suggested that CAP value has a greater ability than other obesity markers to predict IR.

Recent investigations have revealed probable mechanisms connecting hepatic steatosis and IR in obesity. Excessive fat intake triggers inflammation in the body through two primary pathways, which impact insulin signaling. One is the recruitment of macrophages into adipose tissue, and then adipose tissue macrophages polarize to pro-inflammatory states [29, 30]. Another one, excess reactive oxygen species and pro-inflammation can result from deregulated organelles, such as mitochondria, endoplasmic reticulum, and lysosomes, caused by lipotoxicity from ectopic fat [5, 31]. Long-term low-grade systemic inflammation prevents the action of insulin in the insulin signaling pathway and leads to systemic IR. Notably, several studies indicated that fat diet-induced hepatic steatosis perpetuates IR through impaired post-receptor insulin signaling before and independent of the development of obesity. One study indicated that elevated circulating glucose and insulin levels in individuals with MASLD stimulate hepatic de novo lipogenesis, resulting in an increase in IHTG [9]. Moreover, excess systemic free fatty acids and dietary lipids enter inside the cells of non-adipose organs such as the liver, muscle, and pancreas, and are deposited as ectopic fat, causing hepatic steatosis [5, 32]. Hepatic steatosis can cause hepatic IR by triggering gluconeogenesis and activating protein kinase (PKC)-epsilon and Jun N-terminal kinase (JNK) 1 [33, 34]. The correlation between CAP values and HOMA-IR proved to be significantly stronger than other obesity markers. In conclusion, IR is the primary pathophysiological abnormality of hepatic steatosis, which is then worsened by hepatic steatosis.

There are several strengths in our study. First of all, this is the first analysis to investigate the association of CAP value and HOMA-IR in the population with overweight or obesity. Second, this study found that CAP value was positively associated with HOMA-IR, the strength of which was much stronger than BMI, VFA, and BFM. Besides, there were still some limitations of our study. Firstly, due to the cross-sectional design, we cannot make a clear description of the causal inferences. We will conduct some cohort studies in the future to confirm the causal relationship between CAP value and IR. Secondly, this study did not assess hepatic fat accumulation with gold-standard imaging such as MRI-PDFF and IR with gold-standard testing such as euglycemic insulin clamp. Thirdly, this study was a one-center study with a limited sample size that only involved the population with overweight or obesity in China. Further studies are required to determine the applicability of this result to populations in other countries or races. Finally, as this was an observational study, unmeasured confounders may have influenced the correlation between CAP value and IR.

## Conclusion

Hepatic CAP value is positively associated with IR in a Chinese population with overweight or obesity. In addition, the correlation between CAP value and HOMA-IR was the most significant of other obesity markers, including BMI, VFA, and BFM. This implies that individuals with high levels of CAP value should be closely monitored for insulin levels to prevent the onset of metabolic syndrome and other complications. Hepatic CAP value may be used as a cost-effective and accessible marker for monitoring and assessing IR.

### Supplementary Information

Supplementary Material 1

## Data Availability

The data generated during or analyzed during the current study are available from the corresponding author upon reasonable request.

## References

[1] Blüher M (2019). Obesity: global epidemiology and pathogenesis. Nat Rev Endocrinol.

[2] Fahed G, Aoun L, Bou Zerdan M (2022). Metabolic syndrome: updates on pathophysiology and management in 2021. Int J Mol Sci.

[3] Pan X-F, Wang L, Pan A (2021). Epidemiology and determinants of obesity in China. Lancet Diabetes Endocrinol.

[4] Watt MJ, Miotto PM, De Nardo W, Montgomery MK (2019). The liver as an endocrine organ-linking NAFLD and insulin resistance. Endocr Rev.

[5] Ahmed B, Sultana R, Greene MW (2021). Adipose tissue and insulin resistance in obese. Biomed Pharmacother.

[6] Rinella ME, Lazarus JV, Ratziu V (2023). A multisociety delphi consensus statement on new fatty liver disease nomenclature. J Hepatol.

[7] Petersen MC, Shulman GI (2018). Mechanisms of insulin action and insulin resistance. Physiol Rev.

[8] Tanase DM, Gosav EM, Costea CF (2020). The intricate relationship between type 2 diabetes mellitus (T2DM), insulin resistance (IR), and nonalcoholic fatty liver disease (NAFLD). J Diabetes Res.

[9] Smith GI, Shankaran M, Yoshino M (2020). Insulin resistance drives hepatic de novo lipogenesis in nonalcoholic fatty liver disease. J Clin Invest.

[10] Bianco C, Pelusi S, Margarita S (2023). Predictors of controlled attenuation parameter in metabolic dysfunction. United Eur Gastroenterol J.

[11] Sasso M, Beaugrand M, De Ledinghen V (2010). Controlled attenuation parameter (CAP): a novel VCTE^™^ guided ultrasonic attenuation measurement for the evaluation of hepatic steatosis: preliminary study and validation in a cohort of patients with chronic liver disease from various causes. Ultrasound Med Biol.

[12] Gastaldelli A (2022). Measuring and estimating insulin resistance in clinical and research settings. Obes Silver Spring Md.

[13] Magali S, Michel B, de Victor L (2010). Controlled attenuation parameter (CAP): a novel VCTE^TM^ guided ultrasonic attenuation measurement for the evaluation of hepatic steatosis: preliminary study and validation in a cohort of patients with chronic liver disease from various causes. Ultrasound Med Biol.

[14] European Association for the Study of the Liver (EASL) (2016). EASL-EASD-EASO Clinical Practice Guidelines for the management of non-alcoholic fatty liver disease. Diabetologia.

[15] Kozłowska-Petriczko K, Wunsch E, Milkiewicz P (2021). Controlled attenuation parameter in nonalcoholic fatty liver disease: the thresholds do matter. Clin Gastroenterol Hepatol Off Clin Pract J Am Gastroenterol Assoc.

[16] Eddowes PJ, Sasso M, Allison M (2019). Accuracy of fibroscan controlled attenuation parameter and liver stiffness measurement in assessing steatosis and fibrosis in patients with nonalcoholic fatty liver disease. Gastroenterology.

[17] Stine JG, Munaganuru N, Barnard A (2021). Change in MRI-PDFF and histologic response in patients with nonalcoholic steatohepatitis: a systematic review and meta-analysis. Clin Gastroenterol Hepatol.

[18] Wei Y, Liu J, Wang G, Wang Y (2023). Sex differences in the association between adipose insulin resistance and non-alcoholic fatty liver disease in Chinese adults. Biol Sex Differ.

[19] Luukkonen PK, Dufour S, Lyu K (2020). Effect of a ketogenic diet on hepatic steatosis and hepatic mitochondrial metabolism in nonalcoholic fatty liver disease. Proc Natl Acad Sci.

[20] Long F, Bhatti MR, Kellenberger A (2023). A low-carbohydrate diet induces hepatic insulin resistance and metabolic associated fatty liver disease in mice. Mol Metab.

[21] Semmler G, Balcar L, Wernly S (2023). Insulin resistance and central obesity determine hepatic steatosis and explain cardiovascular risk in steatotic liver disease. Front Endocrinol.

[22] Hajifathalian K, Mehta A, Ang B (2021). Improvement in insulin resistance and estimated hepatic steatosis and fibrosis after endoscopic sleeve gastroplasty. Gastrointest Endosc.

[23] Bril F, Barb D, Portillo-Sanchez P (2017). Metabolic and histological implications of intrahepatic triglyceride content in nonalcoholic fatty liver disease. Hepatol Baltim Md.

[24] Lewis GF, Carpentier AC, Pereira S, Hahn M, Giacca A (2021). Direct and indirect control of hepatic glucose production by insulin. Cell Metab.

[25] Yatsuya H, Nihashi T, Li Y (2014). Independent association of liver fat accumulation with insulin resistance. Obes Res Clin Pract.

[26] Neeland IJ (2019). Visceral and ectopic fat, atherosclerosis, and cardiometabolic disease: a position statement. Lancet Diabet Endocrinol.

[27] Kadowaki S, Tamura Y, Someya Y (2019). Fatty liver has stronger association with insulin resistance than visceral fat accumulation in nonobese Japanese men. J Endocr Soc.

[28] Urata N, Kawanaka M, Nishino K (2020). Fatty liver, and not visceral fat, is more associated with liver fibrosis and diabetes in non-obese Japanese individuals: a cross-sectional study. Life Basel Switz.

[29] Dong B, Zhou Y, Wang W (2020). Vitamin D receptor activation in liver macrophages ameliorates hepatic inflammation, steatosis, and insulin resistance in mice. Hepatol Baltim Md.

[30] Fuchs A, Samovski D, Smith GI (2021). Associations among adipose tissue immunology, inflammation, exosomes and insulin sensitivity in people with obesity and nonalcoholic fatty liver disease. Gastroenterology.

[31] Osborn O, Olefsky JM (2012). The cellular and signaling networks linking the immune system and metabolism in disease. Nat Med.

[32] Hodson L, Rosqvist F, Parry SA (2020). The influence of dietary fatty acids on liver fat content and metabolism. Proc Nutr Soc.

[33] Fang Z, Xu H, Duan J (2023). Short-term tamoxifen administration improves hepatic steatosis and glucose intolerance through JNK/MAPK in mice. Signal Transduct Target Ther.

[34] Mohammad A (2018). Acylated ghrelin induces but deacylated ghrelin prevents hepatic steatosis and insulin resistance in lean rats: Effects on DAG/ PKC/JNK pathway. Biomed Pharmacother.

